# A Molecular Chipper technology for CRISPR sgRNA library generation and functional mapping of noncoding regions

**DOI:** 10.1038/ncomms11178

**Published:** 2016-03-30

**Authors:** Jijun Cheng, Christine A. Roden, Wen Pan, Shu Zhu, Anna Baccei, Xinghua Pan, Tingting Jiang, Yuval Kluger, Sherman M. Weissman, Shangqin Guo, Richard A. Flavell, Ye Ding, Jun Lu

**Affiliations:** 1 Department of Genetics, Yale University School of Medicine, New Haven, Connecticut 06510, USA; 2 Yale Stem Cell Center, Yale Cancer Center, New Haven, Connecticut 06520, USA; 3 Graduate Program in Biological and Biomedical Sciences, Yale University, New Haven, Connecticut 06510, USA; 4 Department of Immunobiology, Yale University School of Medicine, New Haven, Connecticut 06520, USA; 5 Department of Cell Biology, Yale University School of Medicine, New Haven, Connecticut 06520, USA; 6 Department of Pathology, Yale University School of Medicine, New Haven, Connecticut 06520, USA; 7 Interdepartmental Program in Computational Biology and Bioinformatics, Yale University, New Haven, Connecticut 06511, USA; 8 Wadsworth Center, New York State Department of Health, Albany, New York 12208, USA; 9 Yale Center for RNA Science and Medicine, New Haven, Connecticut 06520, USA

## Abstract

Clustered regularly-interspaced palindromic repeats (CRISPR)-based genetic screens using single-guide-RNA (sgRNA) libraries have proven powerful to identify genetic regulators. Applying CRISPR screens to interrogate functional elements in noncoding regions requires generating sgRNA libraries that are densely covering, and ideally inexpensive, easy to implement and flexible for customization. Here we present a Molecular Chipper technology for generating dense sgRNA libraries for genomic regions of interest, and a proof-of-principle screen that identifies novel *cis*-regulatory domains for miR-142 biogenesis. The Molecular Chipper approach utilizes a combination of random fragmentation and a type III restriction enzyme to derive a densely covering sgRNA library from input DNA. Applying this approach to 17 microRNAs and their flanking regions and with a reporter for miR-142 activity, we identify both the pre-miR-142 region and two previously unrecognized *cis*-domains important for miR-142 biogenesis, with the latter regulating miR-142 processing. This strategy will be useful for identifying functional noncoding elements in mammalian genomes.

## Abstract (web summary)

The interrogation of noncoding regions of the genome with CRISPR strategies requires dense and, ideally, inexpensive libraries to ensure coverage and customisation. Here the authors present 'Molecular Chipper' that uses fragmentation to generate guide RNAs from input DNA.

## Graphical abstract

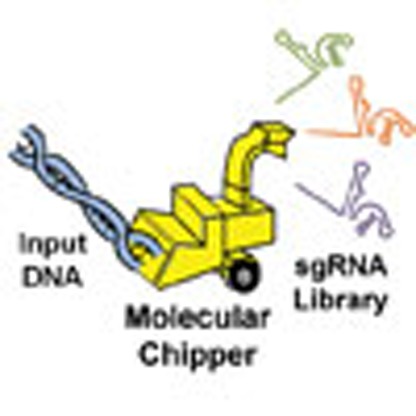

Genome editing using *Streptococcus pyogenes* (sp) Cas9 and single-guide-RNA (sgRNA) libraries is a powerful tool to screen for functional genetic regulators in mammalian cells by generating biallelic loss-of-function sequence alterations^1^,^2^,^3^,^4^,^5^,^6^. Given the protospacer adjacent motif (PAM) of ‘NGG' for Cas9, it is theoretically possible to have one sgRNA every ∼8 bp on average, thus raising the possibility of using high-density tiling sgRNA libraries for functional interrogation of noncoding genomic regions. Indeed, Canver *et al.*^7^ recently demonstrated that *cis*-regulatory elements for BCL11A can be identified using computationally designed clustered regularly-interspaced palindromic repeats (CRISPR) libraries of ∼1,300 sgRNAs for selected enhancer regions. Several sgRNA libraries for protein-coding genes and/or limited numbers of noncoding genes have been reported^2^,^3^,^4^,^5^,^7^,^8^, which were produced by careful bioinformatics design, oligonucleotide synthesis on microarray and cloning of oligonucleotide pool(s) into vectors. This approach has been very useful, but requires computational expertise for genome-wide sgRNA design and expensive microarray synthesis, and thus is challenging for most laboratories. Importantly, without prior knowledge of the locations of critical noncoding-element-containing regions, functional mapping of noncoding genomic regions requires sgRNA libraries that densely populate regions of interest, and the ideal method requires flexibility for adjusting the scale of sgRNA production to easily cope with this need.

MicroRNAs (miRNAs) are an important class of noncoding genes that regulate diverse biology. miRNAs are transcribed as primary transcripts that undergo sequential processing into pre-miRNAs and mature miRNAs^9^. miR-142 is abundantly expressed and plays critical roles in haematopoietic cells and beyond^10^,^11^,^12^,^13^. In addition, somatic miR-142 mutations have been identified in haematopoietic malignancies^14^,^15^, with mutational patterns suggestive of a haploinsufficient tumour suppressor^14^. Moreover, miR-142 expression is frequently downregulated in chronic myelomonocytic leukaemia^16^, further underscoring the importance of maintaining the correct expression level of this miRNA. However, molecular regulation of the expression of this miRNA is poorly understood and *cis*-domains important for miR-142 processing have not been characterized.

In this study, we report a Molecular Chipper approach to generate a near-base-resolution sgRNA library densely covering input DNA piece(s). Using this approach, we generated a sgRNA library for 17 miRNA-containing regions. We utilized this library and a reporter cell line in an enrichment screen to identify *cis*-regulatory elements for murine miR-142 biogenesis. We report two novel noncoding *cis*-regions that control miR-142 processing, thus providing a proof of principle of using a Molecular-Chipper-generated library for functional screen of important noncoding elements.

## Results

### The Molecular Chipper approach for sgRNA library generation

**Figure 1:**
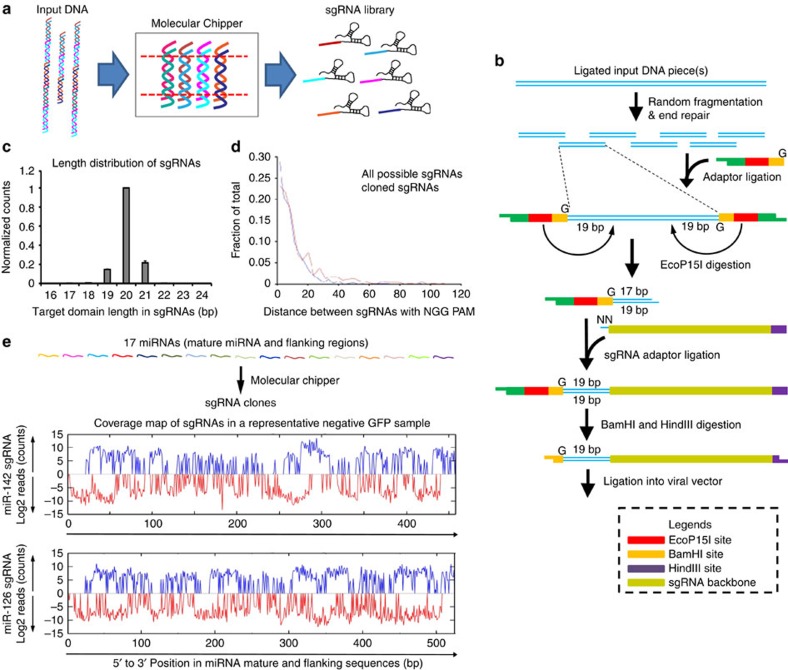
Cloning of a miRNA sgRNA library using the Molecular Chipper method. (**a**) Overview of the Molecular Chipper method to generate a sgRNA library from pieces of input DNA. (**b**) Detailed schematics of the Molecular Chipper procedure. Briefly, an EcoP15I-site-containing adaptor is ligated to randomly fragmented DNA ends, and enzymatically released 20 bases (a G base plus 19 bases from ends of DNA fragments) are cloned as a pool into a viral vector. (**c**) Seventeen murine miRNAs (or miRNA cluster) and their flanking genomic sequences were used to generate a sgRNA library. Length distribution of the targeting portions of sgRNAs within the library is shown. Note that the length was calculated by one base G (in adaptor) plus the length of random ends of fragments from input DNA. The counts for each length are normalized to those of the 20-base-targeting motif sgRNAs within each biological replicate. Error bars represent s.d. *N*=3 biological replicates. (**d**) The distributions of the distances between neighbouring sgRNAs with NGG-PAM, based on all sgRNAs detected in deep sequencing, are shown (red line). The median neighbour distance is 8 bp. Theoretical distribution assumes all possible NGG-PAM sgRNAs (blue line) are present. (**e**) Top: diagram showing that the 17 murine miRNAs (or miRNA cluster) and their flanking genomic sequences were used to generate a sgRNA library. Bottom: representative graphs of sgRNA counts mapping to the miR-142 region or to the miR-126 region from one out of three neg-GFP samples is shown, with blue and red indicating mapping to sense and antisense strands, respectively. The positions of sgRNAs plotted were only based on positions of the last targeting domain base.

We designed a Molecular Chipper approach, which in essence takes pieces of input DNA and processes them through a molecular machinery to output sgRNAs that densely cover the input DNA (Fig. 1a). Standard molecular cloning techniques and reagents are utilized, thus providing an inexpensive and easily customizable and adaptable method for sgRNA library construction. Specifically, input DNA pieces were fragmented after an optional ligation step, resulting in randomly distributed fragment ends (Fig. 1b). Such ends (19 bp) were then released by the type III restriction enzyme EcoP15I after adaptor ligation, further ligated with the non-targeting portion of sgRNA and finally cloned into a U6-promoter-driven viral sgRNA expression vector (Supplementary Fig. 1a). The targeting domain contains 20 bases (a G and 19 bases from the input DNA). Sp-Cas9 (Cas9) is known to tolerate mismatches at the 5′ end of sgRNA without a significant impact on targeting efficiency^17^,^18^. To confirm this notion, we tested both G+19mer and G+20mer sgRNAs with a mismatch at the G position on their target sequence, and observed robust high-efficiency CRISPR activity (Supplementary Fig. 1c).

As a proof of principle of sgRNA library generation for noncoding regions, we took 17 murine miRNAs or miRNA clusters (with flanking regions; ∼9 kb total length; Supplementary Fig. 1b; Supplementary Table 1) and used the Molecular Chipper to produce a sgRNA library of ∼1.5 million clones (see Methods). To evaluate the complexity and properties of the library, we made pooled virus from this library and infected BaF3 cells, a murine haematopoietic cell line. We deep sequenced the sgRNAs integrated into the genomes of the infected cells. The lengths of the targeting domain of the sgRNAs were predominantly 20 bases, as designed (Fig. 1c). We found a total of 17,246 unique sgRNAs that map to the input DNA sequences, from both sense and antisense strands (for example, Fig. 1e). Given that sgRNAs in the library contain both those that mapped to NGG-PAM target sites and those on non-NGG-PAM target sites, we first evaluated the density of sgRNAs in the library by only considering NGG-PAM sgRNAs that are compatible with wild-type (WT) Cas9. We observed that the distances between neighbouring sgRNAs were close to the theoretical distribution (Fig. 1d), with a median neighbour distance of 8 bp. When considering all sgRNAs in the library, regardless of their PAM sequences, the median neighbour distance is 1 bp. Of note, the above statistics are likely an underestimate of the library complexity (see Methods). These data support a good level of complexity of our library.

### CRISPR screen identifies *cis*-elements for miR-142 biogenesis

**Figure 2:**
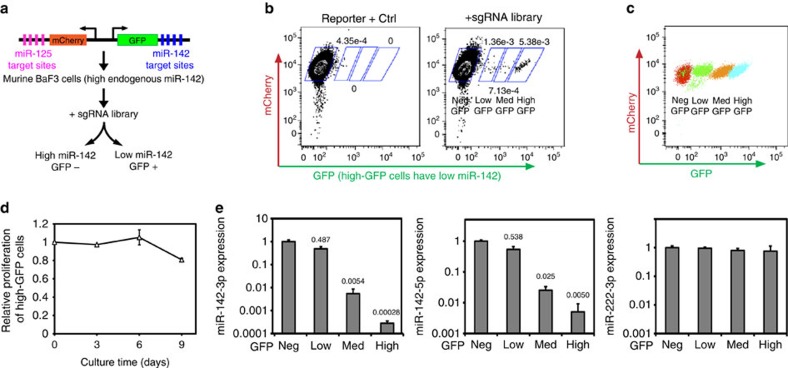
A screen using the Molecular-Chipper-generated sgRNA library to identify both known and unknown functional *cis*-elements for miR-142 expression. (**a**) A diagram showing the miR-142 reporter design and the screen rationale. (**b**) Representative flow cytometry plots (out of three biological replicates) are shown for BaF3 miR-142-3p reporter cells transduced with a control vector or the sgRNA library. Number indicates the percentage of gated population. (**c**) Neg-, low-, med- and high-GFP cells were FACS sorted, and then resorted to improve purify. A representative flow cytometry plot is shown for the four indicated populations after sorting and resorting. (**d**) Competitive proliferation of high-GFP cells and neg-GFP cells was determined. Neg-GFP and high-GFP BaF3 miR-142-3p reporter cells (both mCherry positive) were FACS sorted and mixed with mCherry-negative BaF3 cells. The relative ratio of mCherry-positive to mCherry-negative cells was determined by flow cytometry at the indicated days. Data from high-GFP cells were normalized against those from low-GFP cells. *N*=3 biological replicates. Error bars represent s.d. Note the absence of strong selection against high-GFP cells. (**e**) Mouse miR-142-3p, miR-142-5p and miR-222-3p expression levels in neg-, low-, med- and high-GFP populations (from samples in (**c**)) were determined by qRT–PCR. The relative expression levels are labelled relative to that in neg-GFP samples. Note that data are shown in log scale. Also note that the miR-222-3p expression is shown as a control. *N*=3 technical replicates. Error bars represent s.d. Data are from a representative experiment out of two performed.

We performed a functional screen to identify *cis*-elements in control of miR-142 biogenesis, which is based on the principle that sgRNAs disrupting important elements for miR-142 expression can lead to changes in a reporter for miR-142 activity. We generated a miR-142-3p reporter cell line with constitutive WT Cas9 expression. This reporter cell line was derived from BaF3, which has high endogenous miR-142-3p expression and low miR-125-family miRNA expression^19^. BaF3 cells were transduced with a dual-miRNA reporter construct, with green fluorescent protein (GFP) expression controlled by four miR-142-3p-binding sites in the 3′-untranslated region (UTR), and mCherry controlled by miR-125a activity (Fig. 2a). The resultant BaF3 miR-142-3p reporter line had high mCherry expression and very low GFP levels (referred to as neg-GFP; Fig. 2b, left panel). Thus, sgRNAs that disrupt endogenous miR-142 expression will lead to GFP^+^ cells.

We then transduced the sgRNA library into the reporter cell line with three independent biological replicates, with an infection titre of ∼30% to minimize multiple viral integrations into the same cell. Indeed, GFP^+^ populations emerged in library-transduced reporter cells, which were fluorescence-activated cell sorting (FACS) sorted or double sorted into four fractions based on GFP levels (Fig. 2b,c). Of note, high-GFP cells did not show major competitive proliferative disadvantage in culture compared with neg-GFP cells (Fig. 2d), supporting that the loss of miR-142 expression and high GFP levels do not strongly impact BaF3 cell proliferation and/or survival. Compared with neg-GFP cells, low-, med- and high-GFP cells showed ∼2-fold, >100-fold and >1,000-fold reduction in miR-142-3p levels, respectively (Fig. 2e). Thus, low-GFP cells represent partial disruption of miR-142-3p expression, whereas both med- and high-GFP cells represent near-complete ablation.

**Figure 3:**
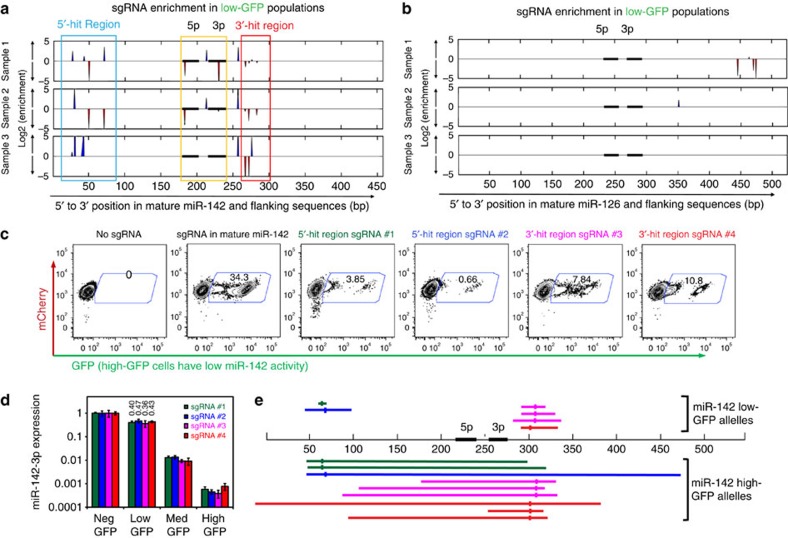
Identification and validation of the 5′- and 3′-hit regions of miR-142. (**a**) Log2 enrichment of sgRNAs in low-GFP cells versus neg-GFP cells is shown for miR-142 in biological triplicates. *X* axis indicates position in bp. Horizontal black bars indicate the locations of mature miRNAs. Blue and red indicate enriched sgRNAs that were mapped to sense and antisense strand, respectively. Note that the positions of sgRNAs plotted were based on positions of the last targeting motif base. Blue and red boxes indicate 5′- and 3′-hit regions. (**b**) Log2 enrichment of sgRNAs in low-GFP cells versus neg-GFP cells is shown for miR-126, as a control, in biological triplicates. (**c**) Single sgRNAs from the hit regions were transduced into BaF3 miR-142-3p reporter cells. The distribution of GFP levels was determined by flow cytometry. Representative flow cytometry plots are shown, with numbers indicating the percentage of cells within the gate. Note that five single sgRNAs were tested and colour coded in the figure, including one from mature miR-142-5p region, two in the 5′-hit region and two in the 3′-hit region. (**d**) Mouse miR-142-3p expression levels in neg-, low-, med- and high-GFP populations sorted from reporter cells transduced with the four single sgRNAs (as in **c**) were determined by qRT–PCR. The relative expression levels were normalized to that in neg-GFP samples. Note that data are shown in log scale. *N*=3 technical replicates. Error bars represent s.d. Data are from a representative experiment out of two performed. (**e**) Low-GFP and high-GFP populations transduced with the four sgRNAs were sorted, and genomic DNA was PCR amplified around miR-142 locus and TA cloned. The deletions in low-GFP (top) and high-GFP (bottom) cells are shown within a schematic diagram depicting the miR-142 locus. Horizontal black bars represent mature miR-142 miRNAs. Deletion alleles are colour coded as in **c**, with short vertical bars in deletion regions indicating the positions of sgRNAs. Positions of sgRNAs correspond to the positions of the last base in the targeting domain.

To identify sgRNAs that disrupt miR-142 expression, we compared the levels of sgRNAs in the three GFP+ populations versus those in neg-GFP cells, and calculated enrichment scores separately for each biological replicate to reflect sgRNA overrepresentation in GFP^+^ cells (Fig. 3a,b; Supplementary Fig. 2a,b; Supplementary Table 2). Several sgRNAs that map to pre-miR-142 (including mature miR-142-3p, miR-142-5p, and the loop region between the two mature miRNA strands) were strongly enriched in high- and/or med-GFP populations across two replicates or more, whereas little or no consistent enrichment was seen for sgRNAs mapping to other miRNAs (for example, Supplementary Fig. 2a,b). Since pre-miRNA regions give rise to mature miRNAs, these data support that known functional elements for miR-142 expression can be identified by the screen.

In addition to the pre-miR-142 region, we observed enriched sgRNAs clustered in regions 5′ and 3′ to mature miR-142 strands in low-GFP samples and, to some degree, in med- and high-GFP samples (Fig. 3a,b; Supplementary Fig. 2a,b), suggesting these harbour potentially unknown *cis*-regulatory domains for miR-142 expression. We refer to them as 5′- and 3′-hit regions. Importantly, the enrichment of a cluster of sgRNAs of different sequences in close sequence proximity not only suggests that the underlying regions are functionally relevant, but also argues against the enrichment being completely driven by off-target effects of sgRNAs. We reasoned that such clustered hits can be a key feature of a high-density sgRNA screens on noncoding regions. Thus, we designed an algorithm, Enriched SgRNA Cluster Scanner (ESCScanner), to capture such clusters. ESCScanner (Supplementary Fig. 3a) examines moving windows along sequences of interest, estimates the probability of observing enriched sgRNA clusters in each window and plots such probabilities at the window locations along sequences of interest (see Methods for details). When applying ESCScanner to our data, we observed consistent cluster enrichment in 5′- and 3′-hit regions, and in pre-miR-142 in all three biological replicates (Supplementary Fig. 3b-c). In the raw enrichment data (Fig. 3a,b; Supplementary Fig. 2a,b), we observed both those sgRNAs that were independently identified as enriched across two or more biological replicates and those appearing in a single replicate, with the latter likely reflecting assay variation. Compared with the raw enrichment, ESCScanner results were more consistent among different biological replicates. Taken together, the data above led us to focus on the 5′- and 3′-hit regions to perform follow-up experiments.

To eliminate the possibility of two sgRNAs getting into the same cell to result in large deletions containing mature miR-142, and to directly validate the two hit regions, we cloned several sgRNA hits and tested single sgRNAs. Each candidate sgRNA led to the appearance of GFP^+^ fractions in BaF3 miR-142-3p reporter cells (Fig. 3c). The low-GFP populations emerged in the presence of these single sgRNAs contained ∼50% miR-142 expression compared with controls (Fig. 3d), similar to levels observed in low-GFP fractions in the presence of the whole sgRNA library (Fig. 2e). Sequencing genomic alleles revealed localized deletions in the 5′- or 3′-hit region in low-GFP cells without affecting mature miRNA sequences, whereas high-GFP cells contained larger deletions that extended into mature miRNA regions (Fig. 3e). The sizes of the deletions, especially in high-GFP cells, tend to be longer than those observed in other cell types^3^,^6^. This may be due to the biological selection of miR-142-low cells and/or due to different cell lines having different intrinsic DNA repair properties. Taken together, these data support that the screen hits can be validated and suggest that the 5-′ and 3′-hit regions quantitatively control miR-142 expression.

### The 5′- and 3′-hit regions regulate pri-miR-142 processing

**Figure 4:**
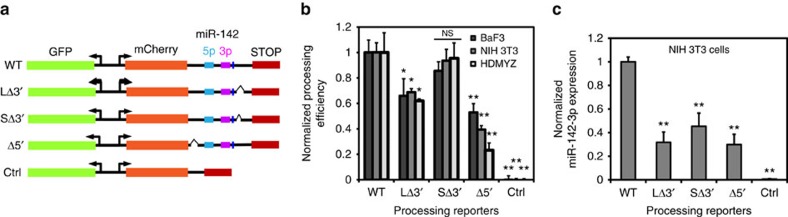
The 5′- and 3′-hit regions of pri-miR-142 regulate miR-142 biogenesis. (**a**) Designs of miRNA processing reporters for control (ctrl), wild-type (WT) miR-142 and its deletion mutants. The narrow vertical blue bar upstream of the 3′-hit region depicts a putative CNNC site, which was not disrupted by the deletions. (**b**) Cleavage efficiencies of the indicated mouse miR-142 processing reporters were determined in the indicated cell lines. *P<0.05; **P<0.01; NS, not significant; Student's *t*-test. *N*=3 biological replicates. Data from a representative experiment out of two performed. Error bars represent s.d. (**c**) NIH 3T3 cells with very low endogenous miR-142 expression were transduced with the indicated mouse miR-142 processing reporters. The expression levels of mature mouse miR-142-3p were determined. **P<0.01; Student's *t*-test. *N*=3 biological replicates. Data from a representative experiment out of two performed. Error bars represent s.d.

The 5′- and 3′-hit regions may regulate miR-142 biogenesis on multiple levels, including transcription and/or processing. Published RNA-seq traces in miR-142 neighbouring regions suggest that the transcription starts >1-kb upstream of 5′ and 3′-hit regions (for example, Supplementary Fig. 4a). Given that sequence elements in primary miRNAs (pri-miRNAs) may control their biogenesis^20^,^21^, we thus tested the possibility that the 5′- and 3′-hit regions regulate miR-142 processing. Taking a widely used strategy for measuring *in vivo* pri-miRNA processing efficiency^21^, we cloned WT or mutant pri-miR-142 sequences in the 3′UTR of mCherry within a mCherry/GFP dual-colour processing reporter (Fig. 4a). The principle of the reporter is that miRNA processing will destabilize mCherry RNA, resulting in a high GFP/mCherry ratio, whereas defective processing can result in a lower ratio. We cloned defined deletions in the 5′-hit region (Δ5′ (66 bp)), and small and large deletions in the 3′-hit region (SΔ3′ (8 bp) and LΔ3′ (23 bp)). Of note, the deletions did not affect a putative CNNC motif (Fig. 4a) previously linked to miRNA processing^20^,^21^. Reporter activities were analysed in murine BaF3, NIH 3T3 and human HDMYZ cells, and in each case, Δ5′ and LΔ3′ resulted in decreased processing efficiency (Fig. 4b). We also examined mature miR-142-3p expression from these constructs in NIH 3T3 cells, which have low endogenous miR-142 expression. Quantitative PCR with reverse transcription (qRT–PCR) confirmed the defective mature miR-142 production from these deletion constructs (Fig. 4c). Deletion of the miRNA hairpin from the reporter constructs largely abolished the reporter activity, as expected (ΔH constructs, Supplementary Fig. 4b,c). In addition, in contrast to Δ5′ and LΔ3′ deletions, another deletion (20 bp) in a region without enrichment (CtrlΔ3′) did not reduce processing efficiency (Supplementary Fig. 4b,c). While we cannot exclude a possibility of 5′- and 3′-hit regions also regulating transcriptional activity of miR-142, we did notice that the deletion of 5′- and 3′-hit regions did not decrease GFP signals in the reporter, which suggests that they were not functioning as enhancer regions in such assays (Supplementary Fig. 4d). Taken together, the data above indicate that the novel 5′- and 3′-hit regions can modulate miRNA biogenesis.

### Compatibility of Molecular Chipper library with mutant Cas9

The library produced by our Molecular Chipper approach includes sgRNAs that map to NGG-PAM sites and non-NGG-PAM sites. While WT Cas9 can only efficiently utilize NGG-PAM sgRNAs, thus resulting in a low percentage of useful sgRNAs in the library, we reasoned that mutant Cas9 with altered PAM specificity could utilize non-NGG-PAM sgRNAs, thus leading to increased utilization of sgRNAs within our library and effectively increasing the density of functional sgRNAs on target DNA regions.

To test this notion, we first introduced a recently reported VQR-mutant Cas9 that can recognize NGA-PAM^22^ into the BaF3 reporter cell line to generate VQR-Cas9 cells, or into BaF3 reporter cells with WT Cas9 to generate cells with both WT- and VQR-Cas9. We then took a single NGA-PAM sgRNA present in our library that mapped to the miR-142 loop region, and tested whether this sgRNA could disrupt miR-142 function. Indeed, we observed the emergence of GFP+ population indicative of low miR-142 expression in VQR-Cas9 only cells, whereas WT Cas9 has a much lower activity with this NGA-PAM sgRNA (Supplementary Fig. 5a,d). To determine the effectiveness of the sgRNA library in the presence of VQR-Cas9, we transduced the library into reporter cell lines expressing either WT Cas9 only, VQR-Cas9 only or both WT Cas9 and VQR-Cas9 (Supplementary Fig. 5b,c,e,f). GFP^+^ cells emerged in VQR-Cas9 cells, albeit to a lower level than in WT Cas9 cells. Library transduction into cells with both WT Cas9 and VQR-Cas9 cells resulted in a reproducibly higher GFP^+^ level than cells with WT Cas9 alone. These data support that our library is also compatible with mutant Cas9 with altered PAM specificity.

## Discussion

In this study, we demonstrate the proof of principle of using Molecular Chipper to generate a high-density sgRNA library and using such a library to identify functional *cis*-regions in miR-142, a noncoding gene. The benefits of the Molecular Chipper approach are the use of standard molecular biology procedures and low cost (as compared with microarray-based oligonucleotide synthesis). It also provides the flexibility to use customizable input DNA as starting materials without the need of complex bioinformatics designs. A recently reported enzymatic method of sgRNA generation produces sgRNAs with >110-bp neighbour distances^23^, which can be very useful in imaging applications, but unlike our approach, cannot be used in mapping functional noncoding elements due to low sgRNA density. In addition to WT Cas9, libraries generated from Molecular Chipper may be used in combination with Cas9 mutants in the future for gene activation/repression or revised to harbour additional sgRNA modules^8^,^24^,^25^,^26^. It is also possible that this approach can be adapted to multiple types of input DNA with longer overall sequence. A computational simulation (see Methods and Supplementary Fig. 6a) suggests that a ∼80-kb input DNA can be used for generating a library with only a slight decrease of sgRNA density, with a similar total bacteria clone number (∼1.5 million) as our current library.

The Molecular Chipper library in this current form of usage has its limitations. Specifically, given the nature of capturing random ends, there will be a large fraction of the library composed of non-NGG-PAM sgRNAs. Although we did observe several hit sgRNAs with a ‘GTGG' PAM sequence (Supplementary Table 2), consistent with WT Cas9 working on some non-NGG-PAMs at lower efficiency^17^,^22^,^27^, most of such non-NGG-PAM sgRNAs cannot be effectively utilized by WT Cas9 and are thus non-functional in screens using WT Cas9. Future improvements of the Molecular Chipper process can be directed at generating PAM-specific sgRNA libraries. On the other hand, such a design may also have its benefits. There are regions in the genome that have >40 bases between neighbouring NGG sequences (for example, see Fig. 1d), which can be thought of as ‘NGG deserts'. It is thus conceivable that the presence of non-NGG-PAM sgRNAs effectively increases the sgRNA density within NGG deserts, as well as the overall density of sgRNAs, as long as such non-NGG-PAM sgRNAs can be functionally utilized. Recent efforts have generated sp-Cas9 mutants with altered PAM specificity^22^. Indeed, we found that our Molecular-Chipper-generated library can be used in combination of VQR-Cas9, which recognizes NGA-PAM and beyond^22^. The biological efficacy of VQR-Cas9 is lower in our experiments than WT Cas9, which could be due to multiple possibilities, such as lower protein expression. Nevertheless, having WT Cas9 and VQR-Cas9 in the same cells increased the overall miR-142-low cells in the screen cell line. We anticipate that further efforts of engineering Cas9 will produce additional Cas9 mutants with varying PAM specificity, which can be utilized with the Molecular Chipper library to increase overall functional sgRNA density for the interrogation of noncoding regions. Alternatively, it may be possible to adapt the Molecular Chipper approach to Cas9 proteins from other species, such as KKH mutant SaCas9 that has a high level of degeneracy in PAM^28^, to further utilize the high-density nature of the sgRNA library. As a second limitation, screens for molecular regulation of gene expression, such as the one performed in this study or the one performed on BCL11A (ref. 7), require good reporters and thus may not be compatible with all genes in the genome. Overall, screens with positive selection tend to be easier than negative selection, and screens using libraries with higher sgRNA content will be more challenging to perform. Nevertheless, we anticipate that Molecular-Chipper-generated libraries can be used in the future to perform gene-expression-based screens of protein-coding gene regulation or in screens with biological selection.

Off-target effects can be a major concern in CRISPR-based screens. One may envision that some sgRNAs mapped to other regions in the genome can target the miR-142 region through off-target effects, thus leading to false-positive results. As another possibility, sgRNAs that map to the miR-142 region may control miR-142 biogenesis through an off-target gene that is responsible for miR-142 production. In our study, we have multiple levels of evidence supporting the specificity of the screen and hits. We show that while plenty of sgRNAs against miR-126 were present in our library, these sgRNAs had little if any effect on miR-142. We further confirmed the hit sgRNAs one by one and used defined deletions of candidate hit regions to demonstrate the validity of the hits. For future screens, how do we minimize the possibility of false positives by off-target sgRNAs? We show that an interesting observation from our screen is the appearance of clusters of enriched sgRNAs at the validated hit regions. Such a feature can be useful to reduce false positives in high-density sgRNA screens, because multiple sgRNAs within a cluster are enriched even though they differ in sequence. We developed an algorithm ESCScanner that can successfully capture such clusters and return more consistent results than simply using raw enrichment data. This algorithm may be useful for future sgRNA screens in noncoding regions, and may further incorporate a feature to flag sgRNAs that may have off-targets. In addition, manually checking hit sgRNAs for potential off-targets may also help to reduce false positives.

Our findings of the *cis*-elements for miR-142 expression can be further studied to determine both their biological functions and regulatory mechanisms in the future. While the screen and follow-up experiments were performed on murine miR-142 sequences, interestingly, we noticed that deletion of the corresponding regions in human miR-142 also reduced its processing activity in a reporter assay (Supplementary Fig. 4e). Given the importance of miR-142 in haematopoiesis and beyond^11^,^12^,^13^,^16^,^29^,^30^,^31^, detailed studies of the 5′ and 3′ regions may yield important insights for disease pathogenesis in haematopoietic malignancies.

## Methods

### sgRNA library construction using Molecular Chipper

The overall procedure of the Molecular Chipper procedure follows the scheme in Fig. 1b, using EcoP15I digestion to obtain random 19mers from input DNA.

As input DNA, genomic DNA fragments of mature miRNA and flanking sequences of 17 mouse miRNAs or miRNA clusters (Supplementary Fig. 1b) were prepared by PCR amplification from previously cloned miRNA expression constructs^19^,^32^,^33^. The miRNA and flanking regions range from 362 to 1,026 bp (Supplementary Table 1). PCR was performed using primers gcctcgatcctccctttatc and aacgcgatcaccactttgta, which are located in the vector sequences outside the miRNA genomic DNA fragments. All PCR products were confirmed by their predicted sizes by running on agarose gels. The PCR products were purified using QIAquick PCR Purification Kit (Qiagen) and pooled together in the same molar ratio. To remove most of extra vector DNA sequences in the PCR products, 80 μg of pooled PCR products were digested with BsrGI, whose sites are immediately flanking the miRNA genomic sequences, followed by gel purification of the DNA fragments ranging 200–1,000 bp using QIAquick Gel Extraction Kit (Qiagen).

We next generated large randomly ligated products before random fragmentation. This step is optional and will not be required if large pieces of input DNA, such as bacterial artificial chromosome clones were used. This step was added to avoid biasing against regions located near the ends of PCR products (because we will perform a size selection after this fragmentation step, and sequences close to the PCR product ends will be represented by very small fragments after fragmentation, and thus will be under-represented in the final library). Specifically, 20 μg of the BsrG1-digested and purified DNA pool were then ligated using 80,000 units of T4 DNA ligase (NEB) in a 1,000-μl ligation reaction for 3 h at 37 °C, followed by ethanol precipitation (add 10% volume of sodium acetate, pH 5.2, and 2 volumes of 100% ethanol; precipitate in −20 °C for 1 h; spin down, wash with 70% ethanol and air dry) and resuspension in water. The sizes of ligation products were checked on agarose gel, which were >10 kb on average. To generate random DNA fragments, 14 μg of the ligated DNA in 120 μl of water were sonicated in a S220 Focused-ultrasonicator (Covaris) for 90 s to result in fragments peaking at sizes of 400–450 bp (peak power=140 V, duty factor=5, cycle/burst=200 and average power=7). Sonicated fragments were repaired by a 150-μl End Repair reaction with 15 μl of the End Repairing Enzyme Mix (NEB), followed by agarose gel purification of the 400–450-bp DNA fragments.

To obtain fragment ends from both the ends of the random DNA fragments, 12 μg of the DNA fragments were ligated with 20,000 units of T4 DNA ligase (NEB) in a 300-μl reaction for 3 h at 37°C, at a ∼1:10 molar ratio to 6.0 μg of an EcoP15I-adaptor that was prepared by annealing two oligonucleotides aaaactcgag**cagcag**tggatccG and/5phos/Cggatcca**ctgctg**ctcgag (IDT). The annealed DNA adaptor contains an EcoP15I site (in bold) followed by a total 8-bp spacer, including a BamHI site (underlined) and a G (capitalized) at the end for later sgRNA cloning. The adaptor-ligated DNA fragments were purified from adaptor monomer and other non-specific bands by running on 1% agarose gels. An amount of 5 μg of the EcoP15I-adaptor-ligated gel-purified DNA was digested by 100 units of EcoP15I enzyme (NEB) in a 300-μl reaction for 1 h at 37 °C. After digestion, EcoP15I digestion reaction was cleaned by phenol/chloroform extraction and ethanol precipitation. Precipitated digestion products were gel-purified (on 4% low-melting-point agarose gel) to obtain a ∼38-bp DNA fragment pool (EcoP15I-adaptor+19/17 bases from ends of random DNA fragments). To ligate to the rest of sgRNA backbone, 280 ng of the purified 38-bp DNA pool was ligated in a 50-μl reaction with 4,000 units of T4 DNA ligase for 3 h at 37°C, at a 1:5 molar ratio to 2.75 μg of an sgRNA backbone adaptor. The sgRNA backbone adaptor contains two Ns for binding to overhangs from EcoP15I digestion products, the remaining sgRNA sequence (without the target recognition domain), a polyT stretch for polymerase III transcriptional termination and an HindIII site for cloning. This sgRNA backbone adaptor was prepared by annealing two oligonucleotides below (IDT), followed by gel purification on 4% low-melting-point agarose gels to eliminate improperly annealed products. /5phos/nngttttagagctagaaatagcaagttaaaataaggctagtccgttatcaacttgaaaaagtggcaccgagtcggtgc-tttttttaagctttat and ataaagcttaaaaaaagcaccgactcggtgccactttttcaagttgataac-ggactagccttattttaacttgctatttctagctctaaaac. The ligated sgRNA DNA pool was cleaned using QIAquick PCR Purification Kit (Qiagen), digested in 50 μl with 20 units each of BamHI and HindIII overnight at 37 °C, purified on 4% low-melting-point agarose gel to obtain a ∼115-bp sgRNA pool. This sgRNA pool was quantified by SYBR Safe Gel Stain (Invitrogen) on a fluorometer, and ligated into BamHI–HindIII sites of a retroviral vector pSUPER-CRISPR (see Constructs), which contains a U6 promoter and a puromycin selection marker. Ligation products were transformed by electroporation into competent NEB5 alpha cells (NEB). Several small fractions of transformation were plated, which led to an estimate of 1.54 million total transformed clones. Transformation culture was grown overnight in 100 ml of LB medium containing 100 μg ml^−1^ of ampicillin for plasmid DNA preparation.

### Constructs

This retroviral pSUPER-CRISPR vector (Supplementary Fig. 1a) for cloning sgRNA library was prepared by cloning the human U6 promoter, followed by BamHI and HindIII sites (for cloning sgRNA library) to replace the H1 promoter through the EcoRI–HindIII sites of retroviral vector pSuper.retro.puro (Oligoengine).

The lentiCas9-Blast construct^34^ was obtained from Addgene.

The miR-142-3p reporter construct was generated based on a bidirectional lentiviral EGFP/mCherry reporter (EGFP miR-T/mCherry miR-T vector^35^, a kind gift from Irvin Chen). The original miRNA target sequences were first removed, and then four copies of miR-142-3p complementary sequences (tccataaagtaggaaacactacacgattccataaagtaggaaacactacaacgcgttccataaagtaggaaacactacatcactccataaagtaggaaacactaca) were inserted in the 3′UTR of EGFP, whereas four copies of miR-125a-5p complementary sequences (taatcacaggttaaagggtctcagggacgattcacaggttaaagggtctcagggaacgcgttcacaggttaaagggtctcagggatcactcacaggttaaagggtctcaggga) were inserted in the 3′UTR of mCherry.

Pri-miRNA processing reporters were constructed by cloning WT or deletion mutants of murine miR-142 plus flanking regions (Supplementary Table S1) in the 3′UTR of EGFP the miR-142-3p reporter construct after deleting miR-142-3p and miR-125a target sequences. A long 23-bp sequence gccacgccgcggccccctgccac (LΔ3′) or a short 8-bp sequence cacgccac (SΔ3′) were deleted in the 3′-hit region, a 66-bp sequence acccacaaggcccagggcgggccctctagggggccacaggcagggtggagcggtccctgggaagtt (Δ5′) was deleted in the 5′-hit region, a 20-bp sequence AATGCACGTCCGTGAGGATA (CtrlΔ3′) was deleted 3′ side of the 3′-hit region and a 85-bp sequence acagtgcagtcacccataaagtagaaagcactactaacagcactggagggtgtagtgtttcctactttatggatgagtgcactgt (ΔH) was deleted in the entire miR-142 hair-pin region to remove the stem loop. The double deletions of ΔH in combination with LΔ3′, SΔ3′ or Δ5′ were also generated. In addition, a control vector without any miRNA fragment in the 3'UTRs was cloned. Human pri-miR-142 processing reporters were constructed by cloning WT or deletion mutants of human miR-142 plus flanking regions, amplified by the oligonucleotide pair atgctgagtcaccgcccaca and ctccccgcccccaaagactgc. A long 24-bp sequence cacgccactgctgccgcccgctgc (LΔ3′) or a short 8-bp sequence cacgccac (SΔ3') was deleted at the location corresponding to the 3′-hit region; a 64-bp sequence gcccacaaggcccagggcgggccctcggggggccctggcagggttggggggatcttaggaagcc (Δ5′) was deleted corresponding to the 5′-hit region.

To validate miR-142 sgRNAs from the screen, a sgRNA targeting the mature miR-142-5p sequence was constructed by T4 DNA kinase phosphorylating and annealing two oligonucleotides, caccgagtagtgctttctactttat and aaacataaagtagaaagcactactc, and cloning into BsmBI sites of construct lentiCRISPRv1 (ref. 34). Similarly, two sgRNAs targeting the 5′-hit region and two sgRNAs targeting the 3′-hit region of mouse miR-142 were cloned using the following oligonucleotide pairs, caccggtcaccacccacaaggccca and aaactgggccttgtgggtggtgacc (sgRNA #1), caccgccacccacaaggcccaggg and aaacccctgggccttgtgggtggc (sgRNA #2), caccgcggagaccacgccacgccg and aaaccggcgtggcgtggtctccgc (sgRNA #3), and caccgagggggccgcggcgtggcg and aaaccgccacgccgcggccccctc (sgRNA #4), respectively. Two sgRNAs (G+19 and G+20) targeting miR-142-5p, followed by the same NGG-PAM site, were cloned using the following oligonucleotide pairs, caccgagtagtgctttctacttta and aaactaaagtagaaagcactactc, and caccgtagtagtgctttctacttta and aaactaaagtagaaagcactactac.

The VQR-Cas9 expression constructs were constructed by first cloning the mutant Cas9 (Addgene #65771) into pDONR221 (Invitrogen), then into the retroviral destination vector pMIRWAY-dsRed^32^,^33^,^36^. The VQR-Cas9 variant was also cloned into the lentiCas9-Blast construct^34^, in which WT Cas9 and the blasticidin resistance was replaced with VQR-Cas9 and zeocin resistance (Zeo). A NGA-PAM sgRNA was constructed by cloning a 19-bp sgRNA sequence tgcactcatccataaagta, targeting the miR-142 loop, into the pSUPER-CRISPR vector.

All cloned inserts in these constructs were confirmed by Sanger sequencing.

### Cell culture

The murine BaF3 haematopoietic cell line was cultured following a published protocol^19^,^32^, with RPMI 1640 medium containing 10% heat-inactivated fetal bovine serum (Life Technologies), 1% of 100 × Pen/Strep/Glutamine (Life Technologies) and 3 ng ml^−1^ of recombinant murine IL-3 (Peprotech). HDMYZ cells were cultured with RPMI 1640 medium containing 10% heat-inactivated fetal bovine serum and 1% of 100 × Pen/Strep/Glutamine^37^. 293 T cells and NIH 3T3 cells were cultured following protocols in American Type Culture Collection (ATCC). BaF3, HDMYZ and 293 T cells were originated from ATCC and obtained from Dr Todd Golub's lab. NIH 3T3 cells were originated from ATCC and obtained from Dr Diane Krause's lab. All cell lines were visually inspected to confirm their expected morphology. BaF3 cells were tested to confirm their dependence on IL-3. Cells were not tested for mycoplasma.

Retrovirus library was prepared by transfecting library plasmids with packaging plasmids into 293 T cells, following our previously published procedures^32^,^33^,^36^. Lentivirus was packaged in 293 T cells following published protocols^32^,^36^,^38^. Viral infection follows previously described procedures^32^,^33^ unless otherwise noted.

The BaF3 miR-142-3p reporter cell line was derived by infecting BaF3 cells with the lentiviral miR-142-3p reporter construct. A single-cell clone was derived after single-cell FACS sorting. This reporter cell line has very low GFP signal (referred to as GFP negative), due to high endogenous miR-142 expression, and high mCherry signal, due to low endogenous miR-125a/b expression.

The BaF3 miR-142-3p screen cell line was derived from the BaF3 miR-142-3p reporter cell line by infection with lentiCas9-Blast, selection with blasticidin (15 μg ml^−1^), and single-cell sorting and cloning. The BaF3 miR-142-3p cell line expressing VQR-Cas9 was derived from the BaF3 miR-142-3p reporter cell line by infection with pMIRWAY-VQR-Cas9-dsRed, and sorted for dsRed+, or by infection with lenti-VQR-Cas9-Zeo construct and selection by zeocin (500 μg ml^−1^).

### Screen for miR-142-biogenesis-regulating sgRNAs

The screen was performed by infecting 10 million cells (BaF3 miR-142-3p screen cell line) with the retroviral sgRNA library, in three biological replicates (on 2 separate days). Each infection replicate was performed by infecting five six-well plate wells, with each well containing 2 million cells, and then combining cells from the five wells after overnight culture. The infection rate was ∼30%. Each infection replicate was diluted to a total of 50-ml culture medium, and cultured in a 150-mm dish for 1 day before puromycin selection (2 μg ml^−1^). Cells were passaged every 2 days (or when necessary) by transferring 5–10 million cells to 50-ml fresh medium for each passage, with puromycin selection.

Cells were FACS sorted 9 days after library infection, based on negative, low, medium and high GFP levels. The sorted GFP-positive populations were resorted after culturing for 3 days, to achieve higher purity (Fig. 2c).

Genomic DNA were extracted from 2.5 million cells of neg-GFP populations, and 6 × 10^4^–5 × 10^5^ cells of low-, med- and high-GFP populations, or 2.5 million unsorted cells from one infection, by proteinase K digestion, phenol/chloroform extraction, ethanol precipitation and resuspension in water. To sequence the sgRNAs integrated into genomic DNA, genomic DNA samples were amplified by PCR using Phusion DNA polymerase (NEB), 200 ng of genomic DNA and the following pair of primers. The sense primer aatgatacggcgaccaccgagatctacactggaaaggacgcgggatcc**G** contains an Illumina adaptor sequence, followed by the library vector sequence (underlined) and a G (bold) that is the first base of transcription. The antisense primer caagcagaagacggcatacgagat**cgtgat**gctatttctagctctaaaac contains an Illumina adaptor sequence, followed by a six-nucleotide library barcode sequence (bold) and sgRNA backbone sequence (underlined). Please see Supplementary Table 3 for library barcodes and sample assignment. For neg-GFP samples and the unsorted sample, 10 PCR reactions (50 μl each) using a total of 1-μg genomic DNA template were pooled to avoid major loss of library complexity. All PCR products were purified from 3% agarose gel, and mixed (100 ng of each neg-GFP PCR products, and 5 ng of each GFP-positive PCR products). The combined sample was sequenced using an Illumina Hi-Seq2000 at Yale Stem Cell Center Genomics Core, using sequencing primer: tggaaaggacgcgggatccg.

To test the compatibility of the sgRNA library in combination of VQR-Cas9 that recognizes NGA-PAM, the BaF3 miR-142-3p cell line expressing VQR-Cas9 and/or WT Cas9 was infected with the library and cultured for 9 days with the same conditions as described above, followed by flow cytometry analysis of 2 million cells for GFP^+^ populations.

### Next-generation sequencing data analyses

Illumina sequencing data were analysed using custom perl and matlab codes (such codes are available upon request). First, the fastq file was converted to fasta file. Second, sequence reads were separated into specific samples based on barcodes (Supplementary Table 3), and sgRNA backbone sequences were clipped off to retain only the targeting portion of the sgRNAs (without first base G, which is in the sequencing primer). Clipping of sgRNA backbone was performed by searching for adaptor sequence using the following ‘GNNNNNNAGCTAGAAATAGC' in which N matches any nucleotide. The six-nucleotide barcode immediately followed this sgRNA backbone sequence. Third, sequences were mapped to the original input DNA sequences using bowtie, allowing either no mismatch (for all following analyses except noted below) or one base mismatch (only for estimation of sgRNA distance).

For sgRNA length distribution analyses, data were based on all sgRNAs detected in the deep sequencing, which was amplified from integrated retrovirus in the genomic DNA.

For estimation of library complexity, mapped unique sgRNAs were counted from all thirteen samples. Of note, the number is likely an underestimation of the real complexity (see ‘sgRNA library complexity and properties').

For enrichment analyses, sgRNA sequence read counts were first normalized based on total mapped read counts in each sample to derive read frequencies. Next, read frequency of every sgRNA within a given GFP-positive population was divided by those of the corresponding neg-GFP sample from the same biological replicate. To avoid division by 0 or log2 operation on 0, the minimal frequency in neg-GFP samples was set to 6.25 × 10^−7^, and minimal frequency in GFP-positive samples was set to 1 × 10^−8^. Log2 enrichment levels were then calculated and plotted. Positions for each sgRNA were represented by the positions of the last base in the targeting section of the sgRNA. If multiple sgRNAs located at the same position (such as with different targeting domain length) were present, the sgRNA with the best enrichment score is shown. To plot the enrichment plot, only enrichment scores above log2 of 0 were shown, and only sgRNAs located in front of NGG-PAM were shown.

To derive candidate sgRNAs that disrupt miR-142 expression, we used the following criteria. (1) The log2 enrichment level is >2 in either high- or med-GFP sample, in at least two biological replicates. (2) Or, the log2 enrichment level is >2 in low-GFP sample, in at least two biological replicates. (3) The sgRNA is located before an NGG-PAM. (4) The sgRNA is located within miR-142 and its flanking regions.

To estimate the distances between NGG-PAM sgRNAs, only sgRNAs with NGG-PAM sequences on either sense or antisense strands were calculated. The distances were defined by the distance between the third last bases of the sgRNA target recognition domains.

### ESCScanner

The ESCScanner algorithm was designed to scan DNA regions of interest for clusters of enriched sgRNAs (Supplementary Fig. 3a) and was implemented using custom matlab codes that are available on request. ESCScanner takes a given window size (a 21-bp window was applied for analysis on our data, which is extending 10 bp on each side of a given nucleotide position) and scans each of the DNA regions of interest (in this case, all 17 miRNA regions that were used as input for the library) with a moving window. For each window, ESCScanner selects a subset of sgRNAs that meet certain criteria (in this case, only NGG-PAM sgRNAs were analysed) and estimates the probability of observing the enrichment pattern associated with these sgRNAs within a given window. The probability was calculated using the multiplication product of probabilities of individual sgRNA enrichments within the window. The probability of enrichment of each individual sgRNA was estimated using normal distribution with 1-normcdf function in matlab, because the enrichment distribution of the majority of NGG-PAM sgRNAs (Supplementary Fig. 6b) was approximately normally distributed.

After the probabilities were calculated for each window, −log10 (probability) was plotted against the window position, which was represented by the position of the centre nucleotide of the window. Of note, for windows close to the end of DNA regions of interest, the same procedure as above was applied, even though effectively a smaller window was used from beginning of the DNA or to the end of the DNA.

### sgRNA library complexity and properties

We demonstrated a sgRNA library produced from ∼9 kb of input DNA, which resulted in ∼1.5 million bacteria clones, and 17,246 sgRNAs by deep sequencing. We discuss below that (1) the number of sgRNAs detected by deep sequencing is likely an underestimate of the real complexity of the library, and (2) the limited number of sgRNAs is likely not due to a methodological limitation, but rather a saturation effect due to input DNA of limited length.

(1) The numbers of unique sgRNAs obtained are likely underestimates of the real complexity, for several reasons. Specifically, we used deep-sequencing results from transduced BaF3 miR-142-3p reporter cells to obtain the number of unique sgRNAs. There are two steps in this procedure that will result in lower complexity estimation. For one, there was likely complexity loss during the viral production process and the infection process. In addition, we used ∼5-μg genomic DNA (from all screen samples) as template for PCR amplification and sequencing. Such an amount of genomic DNA corresponds to ∼500,000–600,000 cells (considering both diploid cells and cells with more DNA content), which is >2.5-fold below our estimation of library clones (∼1.5 million).

(2) To examine a potential saturation effect, we performed computational simulation. Specifically, we randomly selected subsets of mapped deep-sequencing reads from our deep-sequencing results. We then calculated and plotted the number of unique sgRNAs that can be detected versus the number of randomly selected input sequencing reads. Indeed, Supplementary Fig. 6a supports that there is a saturating effect. We took note that 160,000 randomly selected mapped reads can lead to the detection of 12,310 unique sgRNAs, a number representing 71% of 17,246 (which was detected in the library of ∼1.5 million clones and with∼9.8 million mapped reads). In addition, at the level of 160,000 randomly selected mapped reads, the median distance between neighbouring NGG-PAM sgRNAs lengthened from 8 to 10 bp, which is only a modest decrease in sgRNA density. Given that the library has ∼1.5 million bacteria clones, which is >9-fold higher than 160,000 reads above, we thus estimated that at the same level of bacteria clones, we can cover >9-fold longer DNA (>80 kb) with a density of ∼10-bp median distance between neighbouring NGG-PAM sgRNAs.

### Validation of sgRNAs from the screen

Specific candidate sgRNAs (see Constructs) were prepared into lentiviruses, and were used to infect BaF3 miR-142-3p reporter cells (see Cell culture). Single sgRNA viruses were used. After puromycin selection and culturing for a total of 9 days after infection, cells were analysed by flow cytometry to examine efficiency of altering miR-142 reporter level. In addition, low- and high- GFP populations were FACS sorted to prepare genomic DNA. The miR-142 regions were amplified by PCR to obtain an 850-bp fragment by the following primers: catacggctgggaagcac and tctttctgcgtcagttctgttc, and followed by TA cloning (Invitrogen) and Sanger sequencing. The vast majority of alterations were deletions. Two rare cases of insertions in the presence of deletion were not presented in Supplementary Fig. 3b.

### Pri-miRNA processing reporter assay

Lentiviruses were prepared for WT or mutant murine or human miR-142 processing reporters (see Constructs). These constructs were used to infected murine BaF3 (high endogenous miR-142 expression), NIH 3T3 (low endogenous miR-142 expression) cell lines or human HDMYZ cell line (low endogenous miR-142 expression), where indicated in different figures. Each infection was titred to achieve ∼30% infection rate. Cells were analysed by flow cytometry.

Data were analysed with FlowJo software, by first gating on live cells. The geographic mean fluorescence intensities of GFP and mCherry were calculated in GFP+ cells. Processing efficiency was calculated using the ratios of GFP/mCherry in each sample. Data were normalized by setting the mean of GFP/mCherry ratios in miR-142 WT cells as one, and setting the mean of ratios in control vector as zero.

### qRT–PCR

Total RNA were extracted by TRIzol (Ambion). Complementary DNAs were synthesized using miRscript II RT Kit (Qiagen) and qPCR was performed using miRNA primers from Qiagen and the Power SYBR Green PCR Master Mix (Applied Biosystems), on a C1000 Thermal Cycler (Bio-Rad). For measurement of endogenous miR-142, and miR-222 levels in neg-, low-, med- and high-GFP populations, cells used were from the library screen (Fig. 2e) or single sgRNA infected (Fig. 3d), and U6 small RNA levels were used to normalize the data.

For qRT–PCR analysis of exogenous miR-142-3p expression in NIH 3T3 cells, the processing reporters were infected into NIH 3T3 cells to achieve ∼30% infection rates, and GFP+ cells were FACS sorted to extract total RNA. miR-142-3p expression levels were normalized by U6 small RNA levels. Alternatively, normalization was performed using GFP expression levels, which led to similar results.

### Competitive proliferation assay

To evaluate whether loss of endogenous miR-142 may impact proliferation/survival of BaF3 cells, we performed a competitive proliferation assay. High-GFP and Neg-GFP cells were FACS sorted from BaF3 miR-142-3p reporter cell line with a miR-142 targeting sgRNA. High-GFP and Neg-GFP cells were mixed with parental BaF3 cells (without GFP or mCherry), and the ratios between mCherry-positive and mCherry-negative cells were evaluated by flow cytometry at the indicated days after cell mixing.

### Statistical analyses

Student's *t*-test (two-tailed, unpaired, unequal variance) was used unless specified otherwise.

## Additional information

**Accession codes:** Sequencing data have been deposited in GEO (GSE70011).

**How to cite this article:** Cheng, J. *et al.* A molecular chipper technology for CRISPR sgRNA library generation and functional mapping of noncoding regions. *Nat. Commun.* 7:11178 doi: 10.1038/ncomms11178 (2016).

## Supplementary Material

Supplementary Information Supplementary Figures 1-6 and Supplementary Tables 1-3
