# Supplementary material for: A Molecular Chipper technology for CRISPR sgRNA library generation and functional mapping of noncoding regions
Source: Nat Commun. 2016 Mar 30;7:11178. doi: 10.1038/ncomms11178 (PMC4820989; doi:10.1038/ncomms11178)
Supplement: Supplementary Information — Supplementary Figures 1-6 and Supplementary Tables 1-3 [file ncomms11178-s1.pdf]

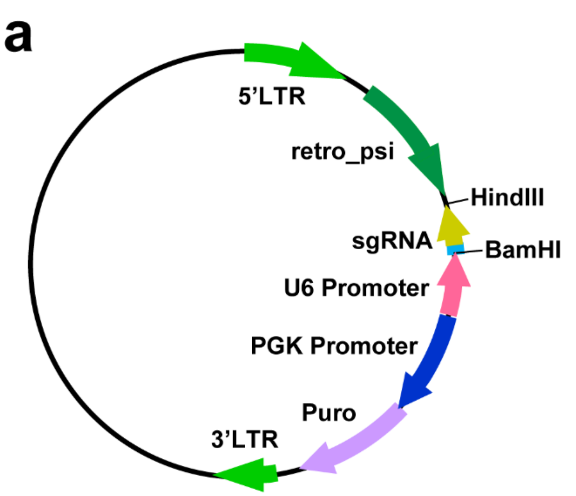

b

miR-16-1
miR-23b
mir-23a_mir-27a_mir-24-2_cluster
mir-24-1
miR-26a-2
miR-26b
miR-27b
miR-29a
miR-29b-1
mir-29b-2_mir-29c_cluster
miR-125b-2
miR-126
miR-142
miR-146
miR-155
miR-222
miR-451

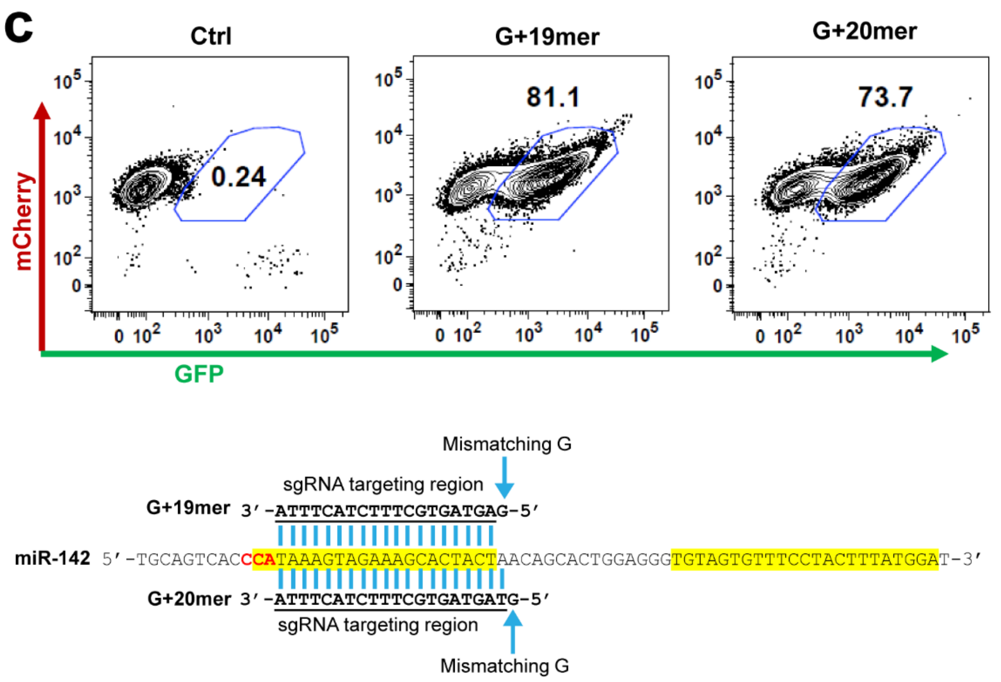

Supplementary Figure 1: sgRNA library generation and the length of sgRNAs for the functional screen.

(a) A diagram of the retroviral vector for sgRNA expression. It contains a U6-promoter-driven sgRNA expression cassette, and a PGK-promoter-driven puromycin resistance gene. Restriction enzyme sites for cloning of the sgRNA library are also indicated. **(b)** A list of 17 mouse miRNA or miRNA clusters included as input for the Molecular Chipper procedure. **(c)** An empty control vector (Ctrl), an sgRNA vector with G+19mer design (G+19) and an sgRNA vector with G+20mer design (G+20) were transduced into the miR-142-3p reporter cell line. Top: representative FACS plot demonstrating efficient inhibition of miR-142 activity. Bottom: Design of the G+19 and G+20 sgRNAs, with miR-142 genomic sequence in the middle. Yellow highlighted regions reflect mature miR-142 miRNA sequences. NGG PAM (CCA on the antisense strand) is shown in red. Note that the first base G in both G+19mer and G+20mer is a mismatch on the target sequence.

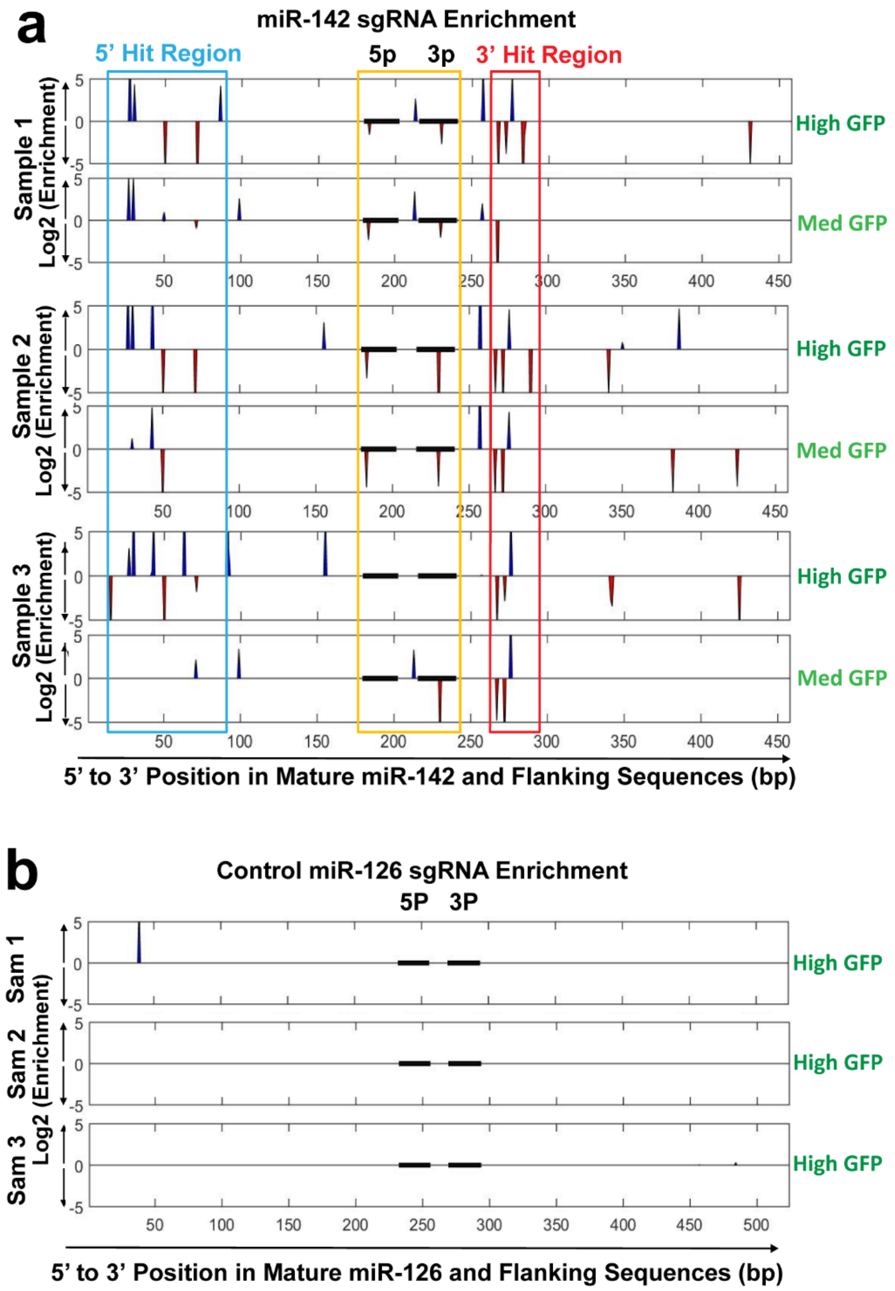

Supplementary Figure 2: Additional data for the sgRNA-based functional screen.

(a) Enrichment plots similar to those in Fig 3a is shown. Left panel, sgRNA enrichments in miR-142 are shown for high- and med-GFP populations (both representing near complete ablation of miR-142 expression) in each of the three biological triplicates. X-axis indicates position in bp. Horizontal black bars indicate the locations of mature miRNAs. Blue and red indicate enriched sgRNAs that were mapped to sense and antisense strand, respectively. The positions of the last targeting-domain base are used to reflect the positions of sgRNAs. Blue and red boxes indicate 5'- and 3'-hit regions. Yellow box highlights sgRNAs mapped to the pre-miR-142 region (including mature miR-142-5p and miR-142-3p, and loop region in between). **(b)** Enrichment plots for sgRNAs mapped to miR-126 from the high-GFP populations are shown, in biological triplicates.

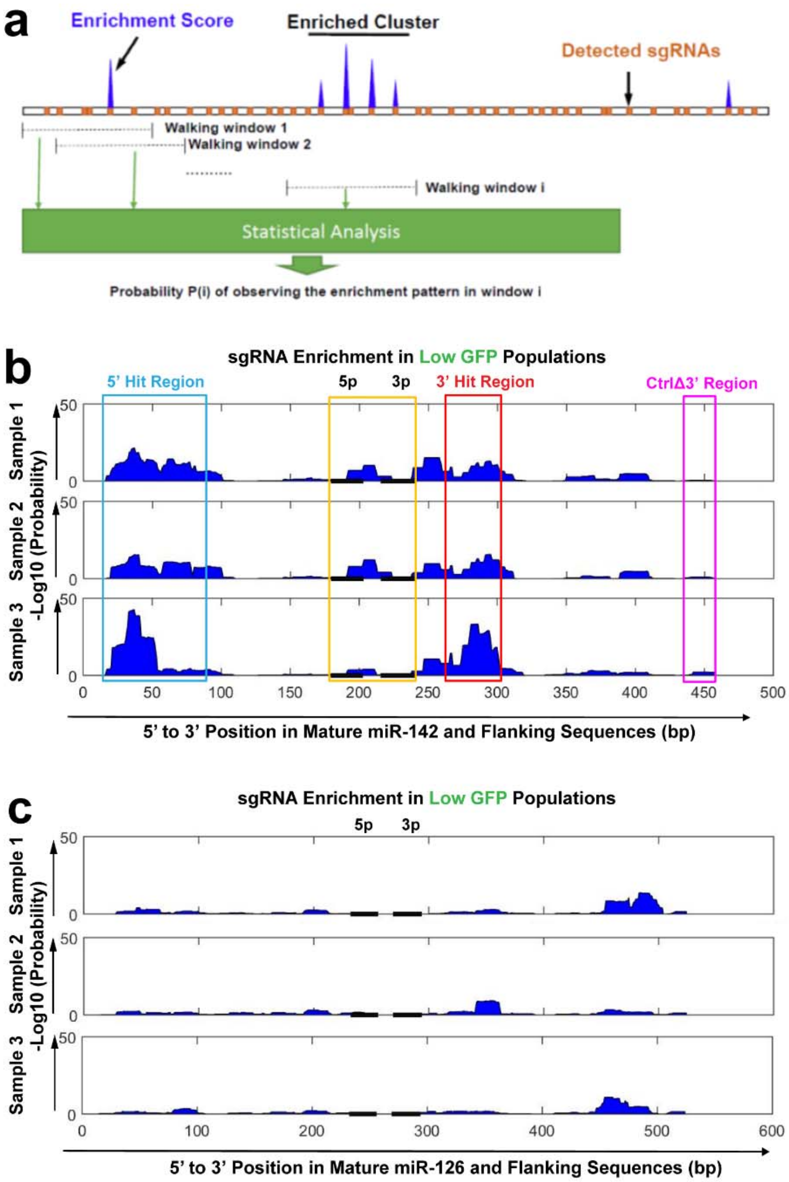

Supplementary Figure 3: ESCScanner analysis of sgRNA enrichment in the functional screen.

(a) Diagram for the principle of the ESCScanner algorithm, which is used to scan for enriched clusters of sgRNAs from the screen. ESCScanner calculates the probability of observing a pattern of sgRNA enrichment within a scanning window. Detected sgRNAs are shown in orange and enriched sgRNAs are shown in blue peaks. **(b)** ESCScanner plots are shown for the miR-142 region for the three biological replicates. Data for the Low-GFP samples are shown. Specifically, a 21-bp scanning window was used and the algorithm was applied independently to the three biological replicates. X-axis indicates the position of the center of the scanning window. Y-axis indicates the –log10 (probability). Horizontal black bars indicate the locations of mature miRNAs. Blue and red boxes indicate 5'- and 3'-hit regions. Yellow box highlights sgRNAs mapped to the pre-miR-142 region (including mature miR-142-5p and miR-142-3p, and loop region in between). Pink box indicates the position of the control 3' deletion (CtrlΔ3') for Supplementary Fig. 4. **(c)** ESCScanner plots are shown for the miR-126 region for the three biological replicates. Data for the Low-GFP samples are shown.

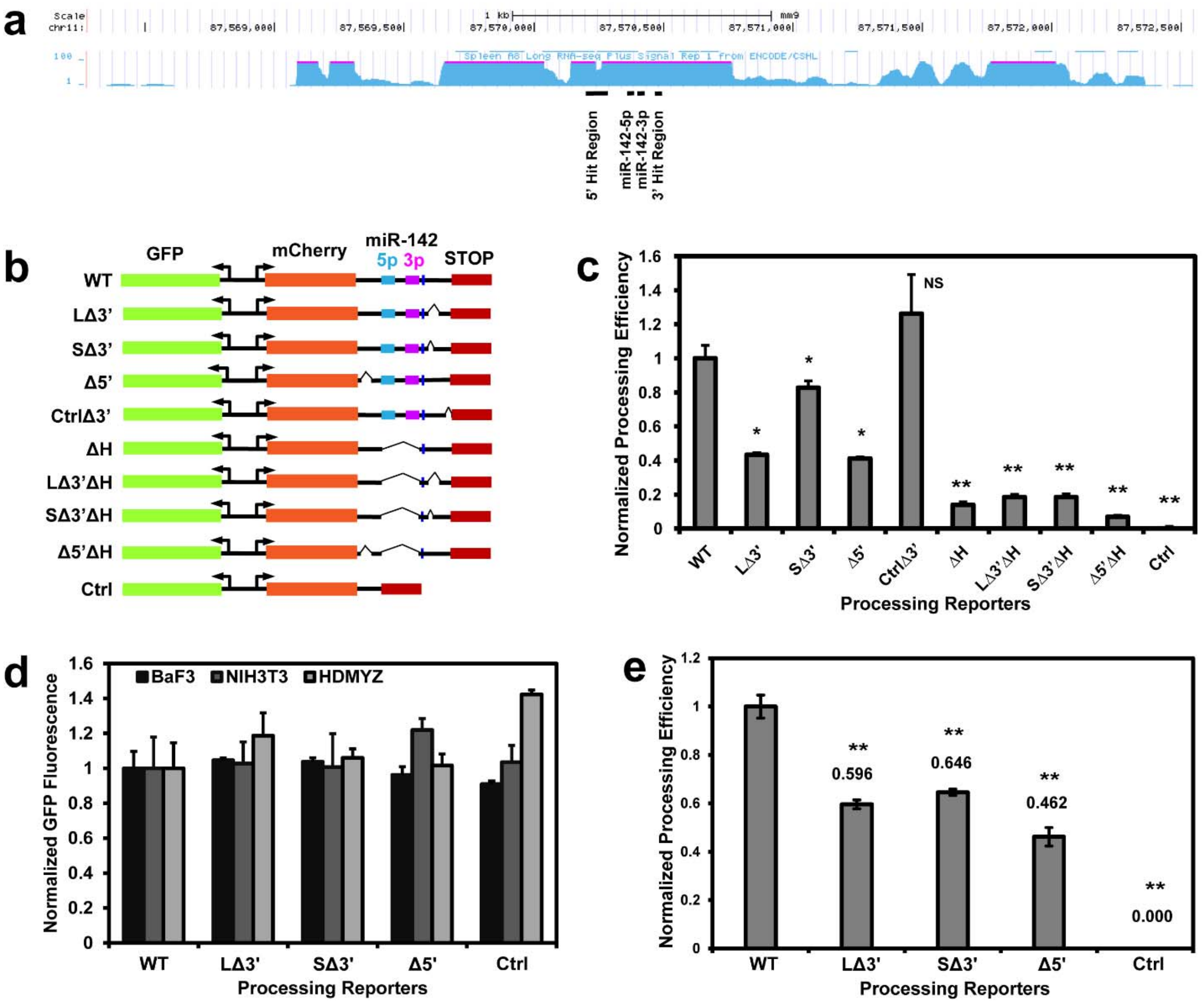

Supplementary Figure 4. miRNA processing reporter activities for additional miR-142 mutants.

(a) A Genome Browser snap shot of RNA seq data of murine spleen cells is shown. Data are from the CSHL long RNA-seq trace. The black bars at the bottom indicate relative positions of the 5'-hit region, miR-142-5p, miR-142-3p and the 3'- hit region. Note that abundant RNA signals can be observed from >1kb upstream of the hit regions on miR-142. **(b)** Designs of additional miR-142 mutants cloned into a miRNA processing reporter. Designs are similar to those in **Fig. 4a**. Additional mutants include a control 3' deletion (CtrlΔ3', 20 bp deletion), deleting hairpin from wildtype pri-miR-142 (ΔH), deleting hairpin from the three mutant pri-miR-142 constructs as in Fig 4a (LΔ3'ΔH, SΔ3'ΔH, and Δ5'ΔH). The narrow vertical blue bar upstream of the 3'-hit region depicts a putative CNNC site, which was not disrupted by the deletions. **(c)** Processing reporter activities of the indicated reporter constructs were determined in the BaF3 cells. * $P<0.05$; **$P<0.01$; NS: not significant. N=3 biological replicates. Data from a representative experiment out of two performed. Error bars represent s.d. **(d)** GFP fluorescence of the indicated processing reporters were determined in the indicated cell lines. N=3 biological replicates. Data were normalized to the mean GFP level in WT construct, and are from a representative experiment out of two performed. Error bars represent s.d. **(e)** Processing efficiencies of the indicated human pri-miR-142 mutant reporters were determined in BaF3 cells. **$P<0.01$; student's t-test. N=3

biological replicates. Data from a representative experiment out of two performed. Error bars represent s.d.

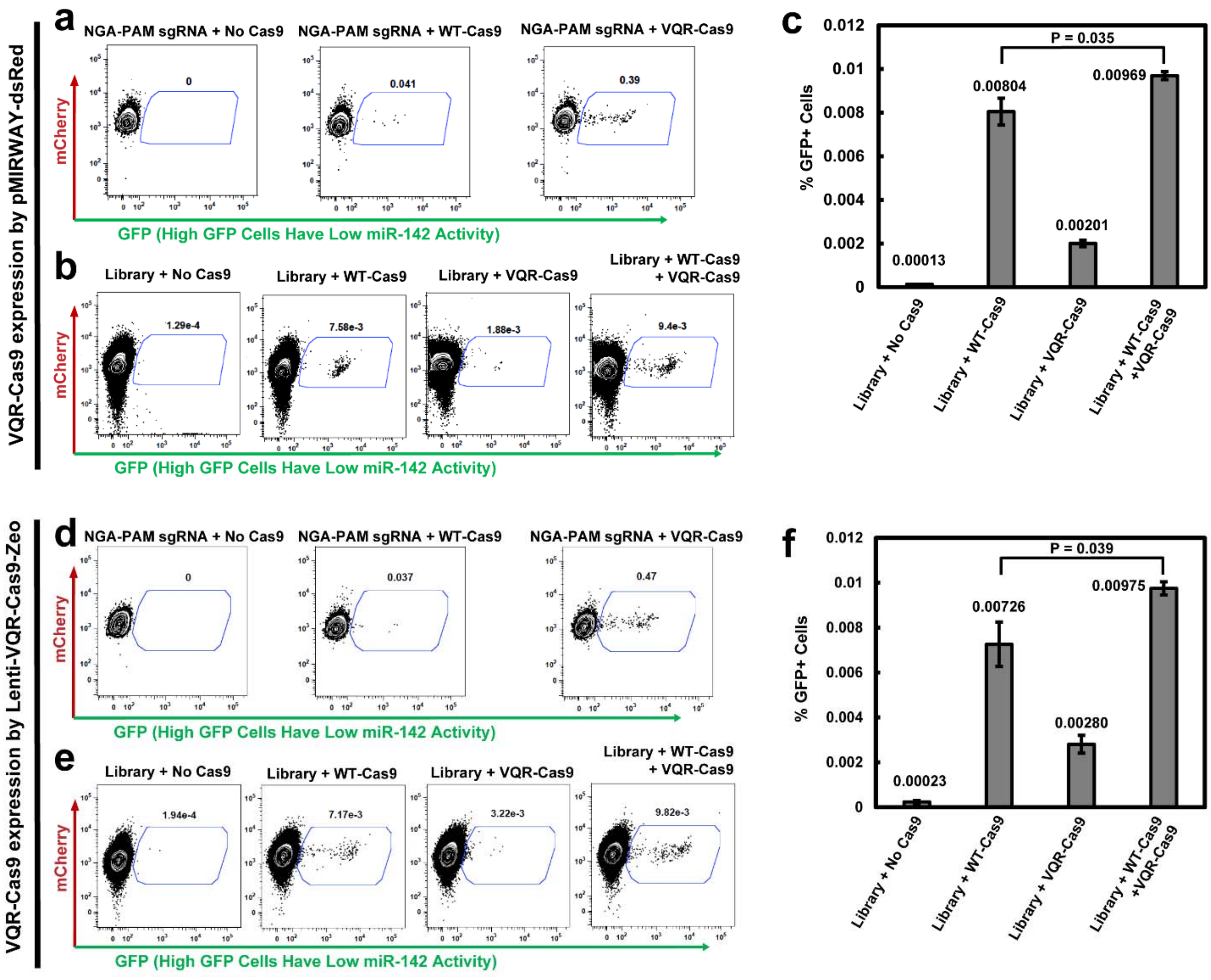

Supplementary Figure 5: Compatibility of Molecular-Chipper-generated sgRNA library with VQR-Cas9.

(a) A NGA-PAM sgRNA in our library, mapped to the miR-142 loop, was cloned and transduced into BaF3 miR-142-3p reporter cells (no Cas9), reporter cells expressing the wildtype Cas9 (WT-Cas9) or reporter cells expressing VQR-Cas9 (with dsRedExpress as selection marker (VQR-Cas9-dsRed)). GFP and mCherry expression levels were determined by flow cytometry. Representative flow cytometry plots are shown, with numbers indicating the percentage of cells within the gates. **(b)** Representative flow cytometry plots are shown for sgRNA library transduced BaF3 miR-142-3p reporter cells (no Cas9), the reporter cells expressing only WT-Cas9, the reporter cells expressing only VQR-Cas9 (with dsRedExpress as a marker), or the reporter cells expressing both WT-Cas9 and VQR-Cas9. Number indicates the frequency of the gated populations. **(c)** Quantified % of GFP+ cells (reflecting low miR-142 activity) for data in **b**. N=3 biological replicates. Error bars represent s.d. Numbers at each bar indicate average % of GFP+ cells. **(d-e)** Experiments similar to (**a-c**) were performed, except that a lentiviral vector with zeocin as selection marker was used. Similar results were observed.

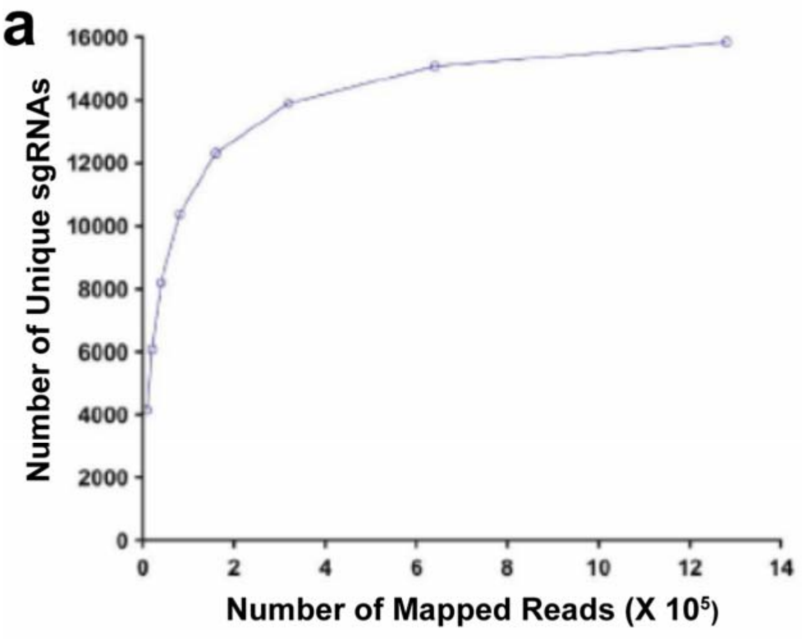

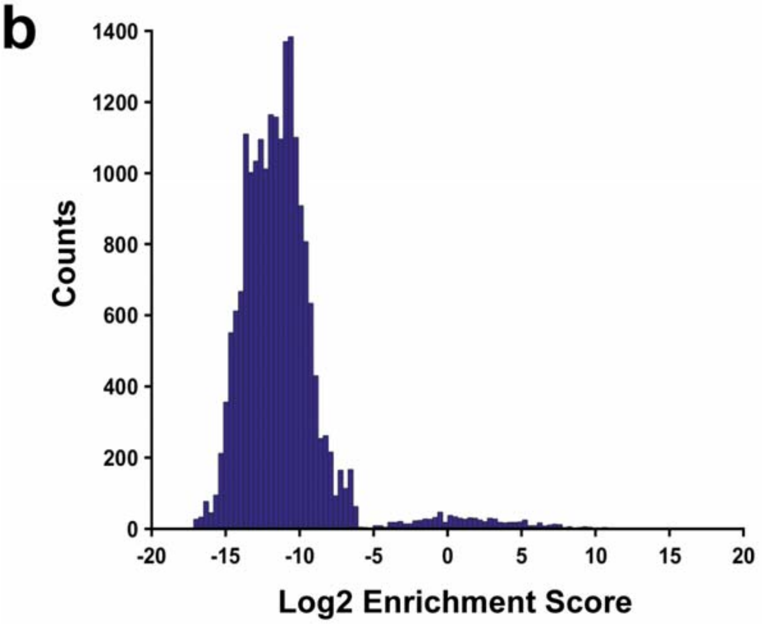

Supplementary Figure 6: Additional data of the sgRNA library complexity and enrichment.

(a) The saturation effect of the Molecular-Chipper-generated sgRNA library. The indicated number of mapped sequence reads were randomly selected from all sequence reads, and the number of unique sgRNAs was determined in each set of randomly selected sequences. Note that 12310 unique sgRNAs were observed with 160,000 randomly selected mapped deep sequencing reads. At this level, the median distance of neighboring NGG-PAM sgRNAs is 10 bp. **(b)** A histogram of the log2(enrichment) scores of all NGG-PAM sgRNAs in our library is shown. Data are based on all experimental pairs of samples (low-GFP, med-GFP and high-GFP versus neg-GFP). There were 2.1% of NGG-PAM sgRNAs with >1 of log2(enrichment).

Supplementary Table 1. Primer Sequences for miRNAs and their Flanking Regions

miRNA construct	Primer sequences to amplify miRNA genomic fragments		Size of PCR (bp)
	Sense	Antisense	
mmu-mir-16-1	TACTATTGAGGTGCTAGGAG	CTAAAACCCATGGCCTTGTG	484
mmu-mir-23b	GGTCCCTAAGGTATTGGTCT	CGGCCATAATTAAAGGGCTG	408
mmu-mir-23a_mir27a_miR-24-2_cluster	TGTGTGGTGAGGTGTACCTA	TTAATGCAAGGGTTACCCGC	780
hsa-mir-24-1	AAACCCAGGTGCATCAAGGA	AACACGTGGCAAATGCTCAG	495
hsa-mir-26a-2	AAGGAACACTTGTGCAGGCT	CTGTGCACTGACAGAAAAGC	485
hsa-mir-26b	TCTGCACTACACCCAGGTT	CAGGAACAGTGGAGGAGGA	467
hsa-mir-27b	GGTTCCTGGCATGCTGATTT	TTCTGTGACTCCCAATACAC	506
hsa-mir-29a	CCTGAAGTAAGTGTCCAGTG	ACACACCCACCATCACTATG	488
hsa-mir-29b-1	GAGGGCTCAGTTACCATTTG	ATGTAGGTCTTCATCCGAGC	471
hsa-mir-29b-2_mir-29c_cluster	ATGTTGAATGGATTTGGTTCTT	TAACAGTACTACATGTCAGAAA	1026
hsa-mir-125b-2	GTTCTGATCTATAGGTGGCC	GCCATATCTTTAGAGTACCG	433
hsa-mir-126	GCTGAGCTGAACATGGAAGA	AGATCCAGGCGCTAGTCAG	524
mmu-mir-142	ATGTTGAGTCACCACCCACA	TATCCTCACGGACGTGCATT	458
hsa-mir-146	CCACATGCCCAGCATGTTTA	CAATCCAACATGACTCCCTC	402
hsa-mir-155	CTGAAGTCTACCTTGCCTTC	CATGTGGGCTTGAAGTTGAG	472
hsa-mir-222	AGGAAGTGAATCTAAAGGTAG	GACTTCATCCTTCATCAAACT	426
mmu-mir-451	GACCTTGGCTGGGATATCAT	TCTTTGGCACAGTGAAGAGG	362

miRNA Constructs: the murine miRNA(s) included as input for sgRNA library production.
Sense: Sequences of sense primer in PCR from murine genomic DNA to amplify the corresponding miRNA.
Antisense: Sequences of antisense primer in PCR from murine genomic DNA to amplify the corresponding miRNA.
Size: Size of miRNA plus flanking regions for each miRNA or miRNA cluster in bp.

Supplementary Table 2. Candidate sgRNAs that disrupt miR-142 expression

						samples								
**sgRNA**	**Gene**	**Strand**	**Position**	**Length**	**PAM**	**Sam1HighGFP**	**Sam1MedGFP**	**Sam1LowGFP**	**Sam2HighGFP**	**Sam2MedGFP**	**Sam2LowGFP**	**Sam3HighGFP**	**Sam3MedGFP**	**Sam3LowGFP**
gtcaccacccacaaggccca	miR-142	1	27	21	NGG	8.005411	0	0	8.765141	5.276985	2.542325	0	0	1.084265
ccacccacaaggcccaggg	miR-142	1	30	20	NGG	4.312409	0	5.527111	4.379338	4.927867	0	3.338627	0	0
cacccacaaggcccaggg	miR-142	1	30	19	NGG	6.232725	1.235737	0	0	0	0	5.263332	0	0
ccagggcgggccctctagg	miR-142	1	43	20	NGG	6.861898	4.800835	0	0	0	1.250508	6.706058	0	9.550644
accgctccaccctgcctg	miR-142	0	50	19	NGG	7.318162	0	2.199697	0.685036	0.131761	4.491559	6.270585	0	0
gaccgctccaccctgcctg	miR-142	0	50	20	NGG	3.785013	7.001066	4.656849	6.740451	0	0.433952	7.309786	0	0
ggaccgctccaccctgcctg	miR-142	0	50	21	NGG	4.72831	0	0	0	0	0	8.885907	0	0
cccctccgtgtaacttccca	miR-142	0	71	21	NGG	2.290962	0	5.266295	9.526861	0.991583	0.023999	1.813299	0	0
ccctccgtgtaacttccca	miR-142	0	71	20	NGG	9.602919	0	0	5.705461	0	0	0	0	0
tagtagtgctttctacttta	miR-142	0	183	21	NGG	1.392517	4.402974	3.989113	0.249048	2.336784	3.677034	0	0	0
cactactaacagcactgga	miR-142	1	213	20	NGG	0	0	2.763994	2.686821	3.424543	2.806901	0	3.2806	0
agtgcactcatccataaagt	miR-142	0	230	21	NGG	10.757184	4.325043	0	0	0	8.031991	0	8.252572	0
gatgagtgcactgtgggctt	miR-142	1	257	21	NGG	2.73708	7.55579	4.943738	7.093705	0	2.060173	0	0	7.242432
atgagtgcactgtgggctt	miR-142	1	257	20	NGG	13.454269	10.589747	0	0	2.00231	3.472131	0.10548	0	0
cggagaccacgccacgccg	miR-142	1	276	20	NGG	4.606831	4.297514	0	5.790099	0	0.397631	7.627885	8.304456	4.831378
agggggccgcggcgtggcg	miR-142	0	267	20	NGG	5.146399	5.679537	0.703752	7.247352	8.631424	1.507806	5.943239	4.823648	7.603144
ggtggcagggggccgcggcg	miR-142	0	272	21	NGG	3.220919	9.216321	3.125319	3.852086	0	0	2.84218	6.94886	5.391727
gtggcagggggccgcggcg	miR-142	0	272	20	NGG	6.663501	0	0	0.904868	0	0.624069	2.692474	5.996965	6.312793
ggggagcctggccaaatga	miR-142	0	341	20	NGG	5.119338	0	0	0	0	0	2.700889	0	0
gcattcgagagagcggctg	miR-142	0	425	20	NGG	0	4.351384	0	0	0	0	6.759109	0	0
gggcaggggcctttattaa	miR-142	1	112	20	NGG	11.330621	0.421764	2.535514	0	2.016078	0.91099	0.175869	0	0
cggagaccacgccacgcc	miR-142	1	275	19	NGG	0.178753	1.762801	0	2.364408	0	0	3.604713	5.017566	1.595674
agaccacgccacgccgcgg	miR-142	1	279	20	NGG	2.851105	0	0	0	0	0	4.034206	0	0
gtggcagggggccgcggc	miR-142	0	273	19	NGG	6.477961	0	0	0	0	0	4.305152	4.98104	4.073721
gaccgctccaccctgcct	miR-142	0	51	19	GTGG	4.342598	8.627086	5.31793	4.51125	0	0	5.993208	0	0
accgctccaccctgcct	miR-142	0	51	18	GTGG	7.149953	0	0	0	0	0	4.457155	0	0

Candidate sgRNAs were derived by the criteria in Methods.

sgRNA: Sequences of sgRNAs, without first base of G. Only sgRNAs located before "NGG" PAM (hiligted in yellow) or "GTGG" PAM (hilighted in blue) are shown.

Gene: The miRNA gene in which the sgRNA is located.

Strand: The strand to which the sgRNA is mapped. "1" stands for "+" strand, "0" stands for "-" strand.

Position: The position of sgRNA relative to the input miRNA sequence. Position is calculated by the position of the last base of the targeting domain in sgRNAs.

Length: The length of sgRNA targeting domain (including the first base of G, which is not shown in the sequence).

Samples: The log2 enrichment level for each sgRNA in each indicated sample is shown. Log2 enrichment level below 0 is shown as 0.

Supplementary Table 3. Samples and Barcodes for Deep Sequencing

Barcode	Primer Barcode	Screen populations
CGTGAT	ATCACG	Sam1NegGFP
CTGATC	GATCAG	Sam1LowGFP
GGACGG	CCGTCC	Sam1MedGFP
CTCTAC	GTAGAG	Sam1HighGFP
CACTGT	ACAGTG	Sam2NegGFP
TTGACT	AGTCAA	Sam2LowGFP
GTAGCC	GGCTAC	Sam2MedGFP
TACAAG	CTTGTA	Sam2HighGFP
TTTCAC	GTGAAA	Sam3NegGFP
CGAAAC	GTTTCG	Sam3LowGFP
CGTACG	CGTACG	Sam3MedGFP
CCACTC	GAGTGG	Sam3HighGFP
GCGGAC	GTCCGC	Unsorted

Barcode: Barcode sequence in sequencing reads.
Primer Barcode: Barcode sequence in reverse PCR primer to amplily sgRNAs for deep sequencing. These barcodes are reverse complement of those listed in "Barcode".
Screen populations: Cells with different GFP levels were sorted and/or re-sorted from the BaF3 miR-142-3p reporter cell line transduced by the sgRNA library in 3 biological replicates, sample 1, sample 2 and sample 3.
